# Co-Delivery System of Vitamin B_12_ and Vitamin E Using a Binary W/O/W Emulsion Based on Soybean Isolate Protein–Xanthan Gum/Carrageenan: Emulsification Properties, Rheological Properties, Structure, Stability, and Digestive Characteristics

**DOI:** 10.3390/foods12234361

**Published:** 2023-12-02

**Authors:** Tian Gao, Xixi Wu, Yiting Gao, Fei Teng, Yang Li

**Affiliations:** College of Food Science, Northeast Agricultural University, Harbin 150030, China; asd002292@163.com (T.G.); 13463261463@163.com (X.W.); g197905312023@163.com (Y.G.); tengfei@neau.edu.cn (F.T.)

**Keywords:** soybean protein, polysaccharide, co-delivery, W/O/W emulsion, digestive characteristics

## Abstract

In this study, the soybean protein isolate (SPI)–xanthan gum (XG) or carrageenan (CA) W/O/W emulsions for the co-delivery of vitamin B_12_ and vitamin E were prepared. The effects of XG and CA concentrations on the physicochemical properties and digestive characteristics of the emulsions were also investigated. The addition of XG and CA improved the SPI aggregation and increased its electrostatic repulsion so that more SPI was adsorbed at the phase interface. The emulsifying activity index and emulsifying stability index increased to 24.09 (XG 0.4%) and 14.00 (CA 0.5%) and 151.08 (XG 0.4%) and 135.34 (CA 0.5%), respectively. The adsorbed protein content increased to 88.90% (XG 0.4%) and 88.23% (CA 0.5%), respectively. Moreover, the encapsulation efficiencies of vitamin B_12_ and vitamin E were increased to 86.72% (XG 0.4%) and 86.47 (CA 0.5%) and 86.31% (XG 0.4%) and 85.78% (CA 0.5%), respectively. The bioaccessibility of vitamin B_12_ and vitamin E increased to 73.53% (XG 0.4%) and 71.32% (CA 0.5%) and 68.86% (XG 0.4%) and 68.74% (CA 0.5%). The best properties of the emulsions were obtained at a 0.4% concentration of XG and 0.5% of CA. This study offers a novel system for delivering bioactive substances, which is favorable for the advancement of food with delivery capability in food processing.

## 1. Introduction

W/O/W emulsion has a multi-membrane and three-phase structure composed of internal water phase (W_1_), oil phase (O), and external water phase (W_2_). It exerts a better effect on the encapsulation and slow release of hydrophilic/lipophilic bioactive substances [1]. This emulsion has great prospects in the food, medicine, and cosmetics fields. Macromolecular hydrophilic emulsifiers can effectively improve the stability of this system [2].

The application of W/O/W emulsion is limited due to the fact that it is a thermodynamically unstable system. Improving the stability of this emulsion has become a research hotspot [3]. Researchers currently prefer to investigate a protein-based W/O/W emulsion with a greater stability. Soybean protein isolate (SPI) is a rich source of high-grade plant protein with good emulsification properties and is often used to prepare emulsions [4]. At present, consumers’ demand for plant-based proteins is beginning to increase. Legumes are widely available and are also a rich source of protein, making the legume-based delivery system a sustainable, high quality, healthful, hypoallergenic, and environmentally friendly ingredient [5]. The high sensitivity of legumes to extreme external environments, such as ions, pH, and temperature, has limited their application in the food industry. Polysaccharides can be easily obtained, have good physicochemical properties, are environmentally friendly, and are widely used [6]. Therefore, they are often used to form more stable complexes with the SPI. These stable complexes are helpful for preparing the emulsion and improving its stability.

Xanthan gum (XG) is a microbial anionic polysaccharide. Because of its molecular structure, XG possesses many excellent properties, including high viscosity, unique rheological properties, and excellent freeze–thaw stability, and is often used in frozen foods [7]. Carrageenan (CA) is a linear sulfated anionic polysaccharide extracted from red seaweed. It has many types because of its structural diversity. It also has emulsification, gelation, thickening, and film-forming properties, and therefore, CA can be utilized in the food field as a gellant, emulsifier, thickener, and suspending agent [8]. Using whey protein and pectin as emulsifiers improved the properties and protection of bioactive substances of the W/O/W emulsion [9]. Klojdova et al. [10] studied CA and showed that it prevented the aggregation of double milk emulsion droplets, thereby preventing coalescence and increasing the encapsulation efficiency. Both Brito-Oliveira et al. [11] and Liu et al. [12] used legume proteins with XG/CA to prepare emulsion gels for the delivery of curcumin.

However, the current research on the characteristics of W/O/W emulsions delivering bioactive substances is more inclined to delivering a single bioactive substance in the W_1_ or O. Therefore, the advantage of these emulsions to simultaneously deliver bioactive substances with different solubilities has not been effectively utilized. Only a few studies have evaluated the possibility of these emulsions as synergistic delivery systems. By using protein fibrils and nanocrystals, Cui et al. [13] improved the structural properties and bioavailability of a W/O/W emulsion encapsulating curcumin and epigallocatechin gallate.

Therefore, this study used XG and CA to form complexes with the SPI and improve the properties of W/O/W emulsions. Then, we investigated the effects of co-delivery of vitamin B_12_ (a hydrophilic bioactive substance) and vitamin E (a lipophilic bioactive substance). Moreover, the stability during storage, pH and thermal stability, the encapsulation efficiency of vitamin B_12_ and vitamin E, and interfacial properties of the SPI-XG and SPI-CA emulsions prepared using the SPI-XG and SPI-CA complexes were compared. The digestive characteristics of these emulsions were further investigated. This study provides a new approach to prepare this novel co-delivery emulsion, which promotes the development of the emulsion.

## 2. Materials and Methods

### 2.1. Materials

Soybean protein isolate, xanthan gum (CAS#11138-66-2), carrageenan (CAS#9000-07-1, intensity 1200), vitamin B_12_, and vitamin E were purchased from Yuanye Biotechnology Co., Ltd. (Shanghai, China). Soybean oil was purchased from a local market (Harbin, China). Distilled water was used, and all other chemicals used were of analytical grade.

### 2.2. Preparation of SPI-XG and SPI-CA W/O/W Emulsions

#### 2.2.1. Preparation of W_1_/O Emulsions

First, 0.2% (*w*/*v*) vitamin B_12_ and 0.1 M NaCl were dissolved in distilled water to prepare the W_1_. Then, 5% (*w*/*w*) PGPR and 2.0% (*w*/*v*) vitamin E were added to soybean oil to prepare the oil phase (O). W_1_ was added to O (10%, *v*/*v*), and they were mixed at 9000 rpm for 4 min using the high-speed shear disperser (IKA, Staufen, Germany) to prepare the W_1_/O emulsion.

#### 2.2.2. Preparation of W_1_/O/W_2_ Emulsions

The W_2_ was prepared by mixing a certain concentration of the SPI solution with different concentrations (0.1%, 0.2%, 0.3%, 0.4%, and 0.5% *w*/*v*) of the XG and CA solutions in a 3:1 ratio. The pH of the SPI, SPI-XG, and SPI-CA solutions was adjusted to 7.00.

Next, W_1_/O was added to W_2_ (40 wt%), and the mixture was homogenized by the same disperser at 8000 rpm for 4 min to prepare the emulsion. The emulsion prepared using the SPI alone served as the control group.

### 2.3. Particle Size and Zeta Potential Measurements

The measurements of the emulsion were conducted using Mastersizer 2000 (Malvern, Worcestershire, UK). The emulsions were diluted. The measurement of the particle size was evaluated through dynamic light scattering. The refractive index of the dispersed phase W_1_/O was set to 1.50 and 1.33 for the continuous phase at room temperature and measured at a fixed detection angle of 90° [4].

### 2.4. Emulsification Properties

The emulsification properties were evaluated using a similar approach of Aewsiri et al. [14]. The measurement was conducted using the UV-2600 ultraviolet–visible spectrophotometer (Shimadzu, Kyoto, Japan). First, the freshly prepared sample was mixed with the SDS solution, and the absorbance values (A_0_) were measured at 500 nm. Then, the emulsion was left undisturbed for 30 min, and the absorbance values (A_30_) were measured again. The emulsifying activity index (EAI) and the emulsifying stability index (ESI) were calculated as follows:

(1) $$\mathrm{EAI}=2\times 2.303\frac{A_{0}N}{C\times \theta \times 10000}$$

(2) $$\mathrm{ESI}=\frac{A_{0}}{A_{0}-A_{30}}\;\times \;30$$

where A_0_ represents the absorbance values at 0 min (500 nm), A_30_ represents the absorbance values at 30 min (500 nm), N is the dilution factor (1000), C is the protein concentration of the W_2_ (g/mL), and θ is the fraction of the oil phase.

### 2.5. Absorbed Protein Content

The emulsion was centrifuged at 10,000 rpm for 15 min. The supernatant was filtered using a 0.22 μm filter, and its protein content was determined [3]. The absorbed protein content (AP) was calculated as follows:

(3) $$\mathrm{AP}(\%)=\frac{C_{t}-C_{s}}{C_{t}}\;\times \;100$$

where C_t_ represents the protein concentration of the emulsion (g/mL), and C_s_ represents the protein concentration of the filtrate (g/mL).

### 2.6. Rheological Properties

Apparent viscosity and frequency sweep of the freshly prepared sample were measured using the RST-CPS rheometer (Brookfield, MA, USA). First, 2 mL of the freshly prepared sample was placed between two parallel plates at room temperature, and 1.0 mm gap was maintained between these two plates. Apparent viscosity was measured with a shear rate range of 0.1–100 s^−1^. Amplitude scanning was performed at 1 Hz to determine the linear viscoelastic region (LVR). Frequency scanning was performed at a constant stress of 1% (within LVR). Changes in the storage and loss moduli (G′ and G″, respectively) were recorded when the frequency was increased from 0.1 to 100 rad/s [15].

### 2.7. Microstructure

#### 2.7.1. Optical Microscope

The freshly prepared sample was diluted with distilled water. Then, 15 μL of the diluted sample was placed on the center of the glass slide and covered with a cover glass. The diluted emulsion on the slide was observed under the BX53 optical microscope (Olympus, Tokyo, Japan).

#### 2.7.2. Confocal Laser Scanning Microscopy

Confocal laser scanning microscopy (CLSM) can better analyze the differences in structures of the emulsions. The CLSM method was slightly modified according to Choi et al. [16]. The images were obtained using the Deltavision OMX SR super-resolution microscope (GE, Boston, MA, USA) in the fluorescence mode. Nile red and Nile blue were dispersed in 10 mL isopropanol to prepare Nile red (1%, *w*/*v*) and Nile blue (0.1%, *w*/*v*) solutions, respectively. Then, the dye solutions were mixed with the emulsion in sequence and stored in the dark at room temperature for 40 min. The excitation wavelengths of CLSM were 510 and 561 nm for Nile blue and Nile red, respectively.

### 2.8. Physicochemical Stability

#### 2.8.1. Storage Stability

First, 10 mL of the freshly prepared sample was added into 15 mL plastic centrifuge tubes, and the heights of the retained emulsion (H_1_) during the storage period and initial emulsion (H_0_) were recorded [17]. Changes in stability are reflected through the creaming index (CI). CI was calculated as follows:

(4) $$\mathrm{Creaming}\;\mathrm{index}\;=\frac{H_{1}}{H_{0}}\times 100\%$$

#### 2.8.2. pH Stability

The pH of the freshly prepared sample was adjusted to 2.0 and 10.0 using 2 M acidic or alkali solutions. Then, the emulsions were stored at room temperature for 3 h. The approach was the same as that of storage stability, which was used to measure the CI of the emulsion.

#### 2.8.3. Thermal Stability

The sample was heated in 50 °C and 90 °C water baths for 30 min. The thermal stability of the sample was determined using the same approach as that of storage stability.

### 2.9. Encapsulation Efficiency

After the free vitamin E in the emulsion was removed with ethanol, the emulsion was centrifuged at 4000× *g* for 30 min. Then, the absorbance of the supernatant was measured at 361 nm. The residue was added to the ethanol and n-hexane mixture (1:2, *v*/*v*), stirred and centrifuged at 7000× *g* for 25 min. Next, the absorbance of the obtained supernatant was measured at 286 nm. Vitamin E and vitamin B_12_ concentrations were determined using the standard curve. The encapsulation efficiencies (EE) of vitamin E and vitamin B_12_ were calculated as follows:

(5) $$\mathrm{EE}(\%)=\frac{C_{1}}{C_{2}}\times 100\%$$

(6) $$\mathrm{EE}(\%)\;=\left(1-\frac{C_{3}}{C_{4}}\right)\times 100\%$$

where C_1_ represents the concentration of vitamin E of the supernatant (g/mL), C_2_ represents the concentration of vitamin E of the emulsion (g/mL), C_3_ represents the concentration of vitamin B_12_ of the supernatant (g/mL), and C_4_ represents the concentration of vitamin B_12_ of the emulsion (g/mL).

### 2.10. Antioxidant Activity

#### 2.10.1. 1,1-Diphenyl-2-picryl-hydrazyl (DPPH) Radical Scavenging Activity

The approach of Huang et al. [18] was slightly modified to evaluate the DPPH radical scavenging activity of the emulsion. Next, 3 mL DPPH solution (0.25 mmol/L) was mixed with 0.3 mL of the sample, which was prepared with anhydrous ethanol. The sample was stored in the dark for 40 min, and its absorbance was measured at 519 nm. The DPPH radical scavenging rate of the samples was calculated as per the following formula:

(7) $$\mathrm{DPPH}\;\mathrm{radical}\;\mathrm{scavenging}\;\mathrm{rate}(\%)=(1\;-\frac{A_{1}-A_{2}}{A_{3}})\;\times \;100\%$$

where A_1_ represents the absorbance of the emulsion, A_2_ represents the absorbance of the emulsion–ethanol solution, and A_3_ represents the absorbance of the DPPH–ethanol solution.

#### 2.10.2. 2,2′-Azinobis-[3-ethylbenzothiazoline-6-sulfonate acid] (ABTS) Radical Scavenging Activity

The method of Yi et al. [19] was slightly modified to evaluate the ABTS radical scavenging activity of the emulsion. Accordingly, the ABTS solution was mixed with an equal volume of potassium persulfate solution. Then, the mixture was stored in the dark at room temperature for 10–18 h to generate the ABTS+ radical. Next, the mixture was diluted with ethanol to produce an absorbance value of 0.70 ± 0.02 at 735 nm. This mixture was used as the working solution for ABTS. Then, 0.3 mL of the emulsion was mixed with 3 mL of the ABTS working solution and stored in darkness at room temperature for 40 min. The absorbance of the emulsion was measured at 735 nm. The ABTS radical scavenging rate of the samples was calculated as follows:

(8) $$\mathrm{ABTS}\;\mathrm{radical}\;\mathrm{scavenging}\;\mathrm{rate}\left(\%\right)=(1\;-\frac{A_{4}-A_{5}}{A_{6}})\times 100\%$$

where A_4_ represents the absorbance of the emulsion, A_5_ represents the absorbance of the emulsion–ethanol solution, and A_6_ represents the absorbance of the ABTS–ethanol solution.

### 2.11. In Vitro Digestion

Gastric digestion: First, 10 mL of the emulsion was mixed with 10 mL of simulated gastric fluid (pepsin, HCl, and NaCl), and the pH of the mixture was adjusted to 2.0. The mixture was incubated on a shaker for 2 h.

Intestinal digestion: First, 10 mL of the gastric digest was mixed with 10 mL of the simulated intestinal fluid (bile salt, trypsin, and lipase), and the pH of the mixture was adjusted to 7.0. The mixture was incubated on a shaker for 2 h. During incubation, the pH of the mixture was maintained at 7.0 with NaOH, with the amount of NaOH solution consumed being recorded.

#### 2.11.1. Release of Free Fatty Acids

The release of free fatty acids (FFAs) during digestion was calculated based on the consumption of the NaOH solution [20]. The FFA release rate was calculated using the following formula:

(9) $$\mathrm{FFA}(\%)=(\frac{M_{\mathrm{oil}}\times V_{\mathrm{NaOH}}\times m_{\mathrm{NaOH}}}{2\times \omega_{\mathrm{oil}}})\times 100\%$$

where M_oil_ is the molecular mass of oil (g/mol), V_NaOH_ is the volume of NaOH solution consumed when neutralizing the released FFA (L), M_NaOH_ is the concentration of NaOH solution (1 mol/L), and *w*_oil_ is the mass of oil in the W/O/W emulsion before digestion (g).

#### 2.11.2. Bioaccessibility

First, 10 mL of the digested sample was centrifuged at 5000 r/min for 40 min. The absorbance of supernatant was measured at 361 nm. Then, 2 mL of the micelles obtained after centrifugation were mixed with 10 mL of the mixture of ethanol and n-hexane (2:1, *v*/*v*). Next, the obtained mixture was centrifugated at 1700× *g* for 10 min. The absorbance of the supernatant was measured at 285 nm. Bioaccessibility can be calculated as follows:

(10) $$\mathrm{Bioaccessibility}(\%)=\frac{C_{5}}{C_{6}}\times 100\%$$

where C_5_ is the concentration of vitamin E/vitamin B_12_ (mg/g), and C_6_ is the total concentration of vitamin E/vitamin B_12_ (mg/g).

### 2.12. Statistical Analysis

All experiments were performed three times, and each result was expressed as the mean ± standard deviation. The statistical significance of the results was determined using SPSS. The data were statistically compared using one-way analysis of variance and Duncan’s test. Differences were considered significant at *p* < 0.05.

## 3. Results and Discussion

### 3.1. Particle Size and Zeta Potential

**Figure 1:**
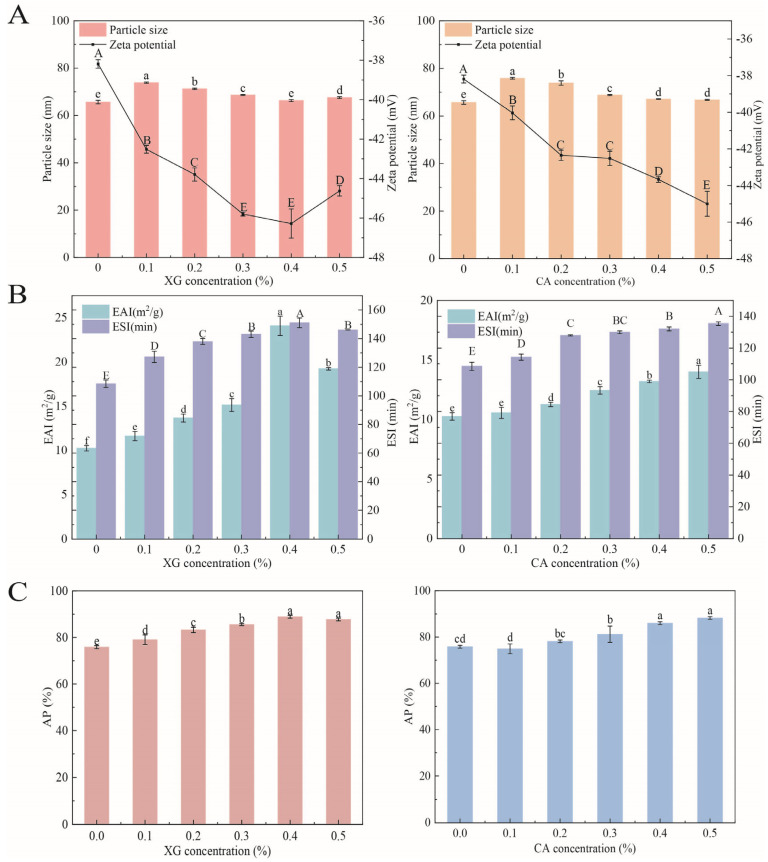
Particle size and zeta potential (**A**), emulsification properties (**B**), and absorbed protein content (**C**) of SPI-XG and SPI-CA emulsions with different polysaccharide concentrations. Different letters in the figures represent significant differences (*p* < 0.05).

Figure 1A presents the particle sizes of SPI-XG and SPI-CA W/O/W emulsions. Compared with that of the SPI emulsion, the particle sizes of the SPI-XG and SPI-CA emulsions increased with the addition of polysaccharides. Although the particle sizes of the SPI-XG and SPI-CA emulsions decreased significantly (*p* < 0.05) with the increased polysaccharide concentration, the particle sizes were not lower than that of the SPI emulsion. The added polysaccharides led to the thickening of the interfacial layer of the SPI-XG and SPI-CA emulsions and the aggregation and bridging flocculation of W_1_/O emulsion droplets, which resulted in their larger particle size. Furthermore, the particle size decreased with the increased polysaccharide concentration, indicating that the polysaccharides adsorbed at the interface prevented oil droplet aggregation, whereas they increased the electrostatic and steric repulsion [4]. The SPI-XG and SPI-CA emulsions with smaller droplets exhibited better stability. However, the particle size of the SPI-XG emulsion increased at 0.5% XG, possibly for two reasons. On the one hand, the high concentration of the SPI-XG complexes increased the droplet diameter. On the other hand, the increased interaction between dispersed molecules adsorbed at the phase interface enhanced droplet interactions [21].

Figure 1A also displays that the absolute zeta potential values of the SPI-XG and SPI-CA W/O/W emulsions increased significantly (*p* < 0.05) after XG and CA were added. The bond between anionic polysaccharides and the cationic side of proteins resulted in the formation of anionic aggregates, thereby forming soluble complexes [22]. The anionic aggregates formed not only made the emulsion droplets negatively charged but also increased the absolute value. Moreover, the adhesion between the SPI-XG complexes attached to the droplet surface caused the bridging flocculation of the emulsion droplets, thereby decreasing the absolute value of the SPI-XG emulsion. Furthermore, because of the large charge density of CA, when the CA concentration increased, a similar increase observed in the CA amount led to a continued increase in the absolute value of the SPI-CA emulsion.

### 3.2. Emulsification Properties

Emulsification properties are crucial indicators of the stability of emulsion and its ability to stabilize the phase interface [9]. The EAI and ESI of the SPI-XG and SPI-CA emulsions increased with an increase in the polysaccharide concentration (Figure 1B). XG and CA contributed to the exposure of more hydrophobic groups of the SPI, improved protein aggregation, and made the SPI generate steric hindrance at the phase interface, which increased the emulsification properties of the emulsions. Furthermore, the EAI and ESI of the SPI-XG W/O/W emulsion decreased at 0.5% XG. Excessive XG may also result in droplet flocculation, an increase in the specific surface area of the droplets, and the competition of the SPI at the phase interface to reduce the amount of SPI adsorbed at the phase interface, thereby decreasing EAI and ESI. The emulsification properties of the SPI-XG emulsion were greater than those of the SPI-CA emulsion. XG had a higher viscosity than CA, which enhanced the continuous phase viscosity, thereby allowing a higher absorption of the SPI at the phase interface and resulting in better emulsification properties.

### 3.3. Absorbed Protein Content

The AP of the SPI-XG and SPI-CA W/O/W emulsions is presented in Figure 1C. Compared with the SPI emulsion, the addition of XG and CA significantly (*p* < 0.05) increased AP, possibly due to the adsorption of more soybean proteins at the phase interface [23]. As the polysaccharide concentration reached 0.5%, AP slightly decreased or increased. This may be caused by the competition between polysaccharides and proteins at the phase interface. Furthermore, the increase in the specific surface area of the droplets reduced the amount of protein adsorbed at the interface. The SPI-XG W/O/W emulsion had a higher AP, indicating that XG can lead to a higher protein adsorption at the phase interface, thus leading to the formation of the denser film at the interface. The results of AP were consistent with those of Section 3.2.

### 3.4. Rheological Properties

**Figure 2:**
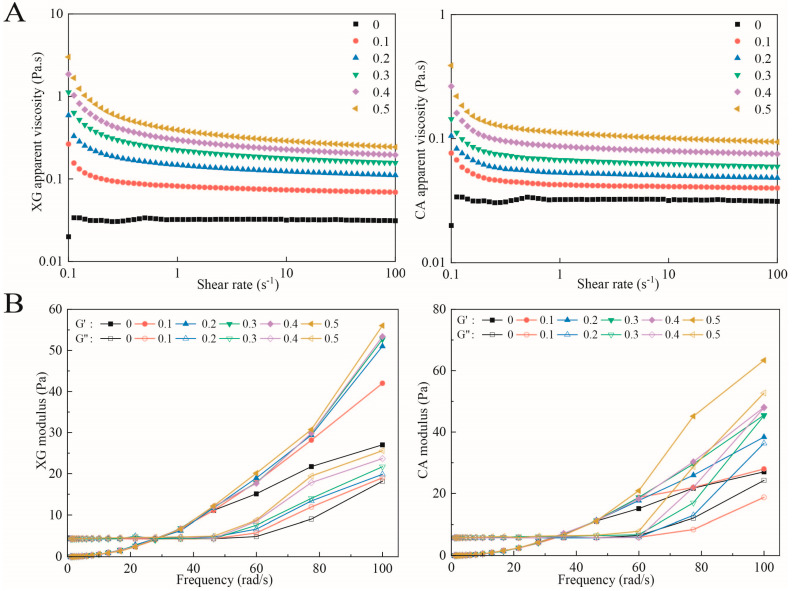
Apparent viscosity (**A**) and frequency sweep (**B**) of SPI-XG and SPI-CA emulsions with different polysaccharide concentrations.

Figure 2A presents the change in the apparent viscosity of the SPI-XG and SPI-CA W/O/W emulsions. The viscosity of the emulsions decreased with the increase in their shear rate. All emulsions exhibited a trend of shear thinning, indicating that all emulsions were pseudoplastic fluids. This may be attributable to the reversible structure formation at some states; thus, the shearing of the material led to structural damage, thereby resulting in a shear-dependent behavior [24]. Furthermore, the viscosity of the SPI-XG and SPI-CA emulsions continuously increased as the XG and CA concentrations increased. The SPI-XG and SPI-CA complexes had higher viscoelasticity, increased viscosity of the continuous phase, enhanced interaction between the droplets, and increased apparent viscosity of the SPI-XG and SPI-CA emulsions. In addition, the viscosity of the SPI-XG emulsion was greater than that of SPI-CA, which may be due to the viscosity of the SPI-XG complexes being higher than that of the SPI-CA complexes.

Figure 2B presents the increase in the storage and loss moduli (G′ and G″, respectively) of the SPI-XG and SPI-CA W/O/W emulsions with the increase in frequency. Furthermore, the values of both G′ and G″ were low and indicate a significant dependence on frequency. The result showed an initial frequency sweep in which G″ was greater than G′ until their intersection was above the specific frequency. This may be attributable to the higher concentration of dispersed phase and the adsorbed biopolymer layers of neighboring droplets with stronger interactions [25]. Beyond this intersection, G′ was higher than G″, which indicated that all emulsions finally exhibited an elastic behavior. In addition, both G′ and G″ of the SPI-XG and SPI-CA emulsions were greater than those of the SPI emulsion, which was attributable to the fact that the added XG and CA enhanced the interaction between the droplets of the SPI-XG and SPI-CA W/O/W emulsions, thereby achieving a more stable network structure [26]. The SPI-CA W/O/W emulsion had a higher modulus and better elastic properties than the SPI-XG W/O/W emulsion.

### 3.5. Microstructure

**Figure 3:**
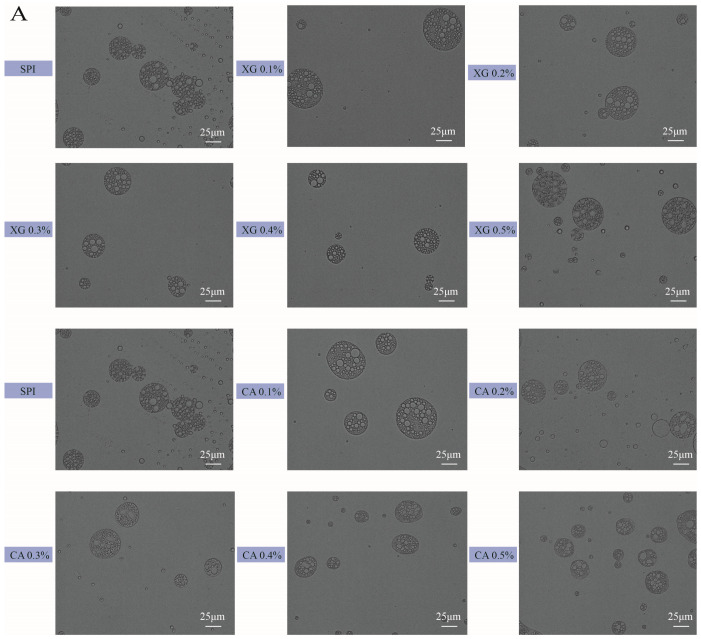

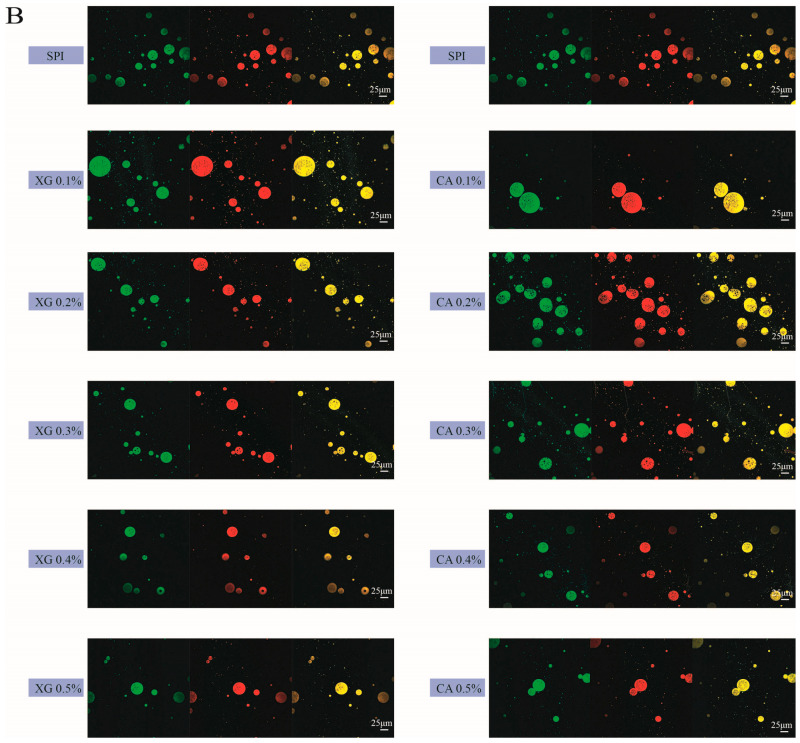
Optical microscopy (**A**) and confocal laser scanning microscopy (**B**) of SPI-XG and SPI-CA W/O/W emulsions with different polysaccharide concentrations. Green represents the SPI, and red represents the oil phase. Yellow represents the composition of the SPI and oil phase.

The optical microscopic images and the CLSM images of the SPI-XG and SPI-CA W/O/W emulsions are presented in Figure 3A,B. The emulsion droplets are spherical, the W_1_ is tightly surrounded by the O, and the W_1_/O emulsion droplets inside these droplets are clearly visible. Meanwhile, the size of the SPI-XG and SPI-CA emulsion droplets were distinct at different polysaccharide concentrations. The size of the SPI-XG and SPI-CA emulsion droplets first increased and then decreased with polysaccharide addition. Compared with the SPI emulsion, the SPI-XG and SPI-CA emulsion droplets were more uniformly dispersed and exhibited almost no aggregation. This may be because the added polysaccharides increased the repulsive forces between droplets and prevented droplet aggregation. However, at a polysaccharide concentration of 0.5%, the size of the SPI-XG emulsion droplets was larger, and the distribution of the SPI-CA emulsion droplets was non-uniform. Excessive polysaccharides could increase the viscosity of the continuous phase of the SPI-XG emulsion, thereby making them more prone to aggregation. The results of microstructure are consistent with those provided in Section 3.1.

### 3.6. Physicochemical Stability

#### 3.6.1. Storage Stability

**Figure 4:**
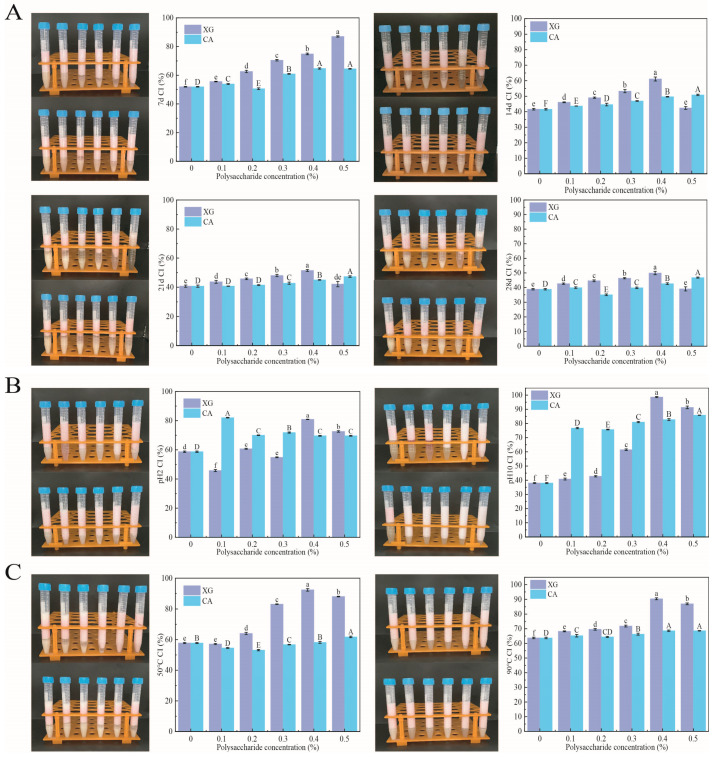
Storage stability at 7 d, 14 d, 21 d, and 28 d (**A**), stability at pH 2 and 10 (**B**), stability at 50 °C and 90 °C (**C**) of SPI-XG and SPI-CA emulsions with different polysaccharide concentrations. Different letters in the figures represent significant differences (*p* < 0.05).

Figure 4A presents the storage capacity of the SPI-XG and SPI-CA W/O/W emulsions. CI represents changes in stability. After the polysaccharides were added, the CI of the SPI-XG and SPI-CA emulsions significantly (*p* < 0.05) increased compared with that of the SPI during the storage period. Although the CI of the emulsion significantly (*p* < 0.05) decreased in the first 14 days, and it slowly decreased in the latter 14 days. This was attributable to the higher viscosity exhibited by the SPI-XG and SPI-CA W/O/W emulsions, which promoted the formation of a more viscous structure of the two types of emulsions and slowed down the movement of W_1_/O droplets, thereby resulting in a more stable emulsion structure [27]. As the polysaccharide concentration increased, the CI of the SPI-XG and SPI-CA emulsions did not decrease as much as that of the SPI emulsion during storage. Meanwhile, excessive polysaccharides significantly (*p* < 0.05) decreased the CI during storage. Therefore, the SPI-XG and SPI-CA emulsions were more stable than the SPI emulsion.

#### 3.6.2. pH Stability

Figure 4B presents the pH stability of the SPI-XG and SPI-CA W/O/W emulsions. The CI of the SPI-XG and SPI-CA emulsions was higher than that of the SPI emulsion at pH 2 and 10. However, the CI decreased substantially at pH 2 because under this condition, the pH value of SPI was below its isoelectric point (IEP), and most of the SPI was protonated. Furthermore, the SPI-XG and SPI-CA complexes were formed through electrostatic interactions. Therefore, at pH 2, the complexes precipitated out of the SPI-XG and SPI-CA emulsions based on strong charge interactions, thereby limiting their molecular absorption at the droplet interface [28]. At pH 10, the emulsions were highly stable because of the stronger electrostatic repulsion within the SPI. The pH value of the SPI was considerably higher than its IEP, which thus lead to the inhibition of the SPI flocculation.

#### 3.6.3. Thermal Stability

Figure 4C displays the thermal stability of the SPI-XG and SPI-CA W/O/W emulsions. First, the trend is broadly the same as that observed in Section 3.6.1 and Section 3.6.2. At 50 °C and 90 °C, the CI of the SPI-XG and SPI-CA emulsions was greater than 50%. Although the emulsion was thermodynamically unstable, the added XG and CA significantly (*p* < 0.05) improved their thermal stability. The thermal stability of the SPI-XG emulsion was greater than that of the SPI-CA emulsion. On the one hand, the higher temperature may have accelerated the motion between the droplets, such that the adsorption ability of these droplets at the interface weakened, making the emulsion unstable. On the other hand, the structure formed by the SPI and CA was not as strong as that formed by the SPI and XG. The degree of structural destruction of the SPI-CA emulsion by higher temperatures was greater than that of the SPI-XG emulsion [29].

### 3.7. Encapsulation Efficiency

**Figure 5:**
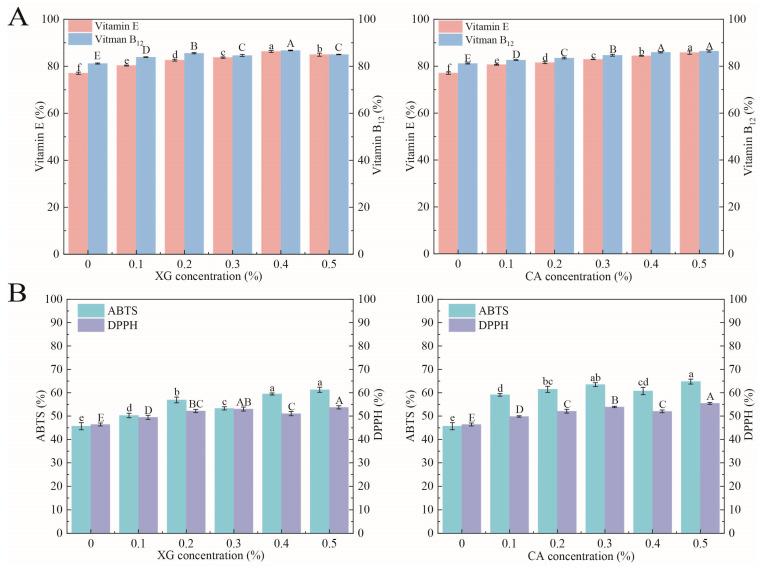
Encapsulation efficiency (**A**) and antioxidant activity (**B**) of SPI-XG and SPI-CA emulsions with different polysaccharide concentrations. Different letters in the figures represent significant differences (*p* < 0.05).

Figure 5A presents the effect of different polysaccharide concentrations on the encapsulation efficiencies of the SPI-XG and SPI-CA W/O/W emulsions. The encapsulation efficiencies of the emulsions for vitamin B_12_ and vitamin E increased from 81.17% and 77.03% (SPI) to 86.72% (XG 0.4%) and 86.47% (CA 0.5%) and 86.31% (XG 0.4%) and 85.78% (CA 0.5%), respectively. The SPI emulsion had the lowest encapsulation efficiency. Furthermore, the encapsulation efficiency of the emulsions was highest when the XG concentration was 0.4% and the CA concentration was 0.5%. The added polysaccharides increased the viscosity of the continuous phase, thereby resulting in a more robust SPI-XG and SPI-CA coating around the W_1_/O droplets [30]. The coating inhibited vitamin B_12_ and vitamin E release and prevented the migration of internal substances, leading to a higher encapsulation efficiency of the whole system [31]. Moreover, a certain decrease in the encapsulation efficiency was observed with an increase in the polysaccharide concentration. At a possibly too high polysaccharide concentration, the complexes caused the flocculation or aggregation of the emulsion droplets, thereby decreasing the stability of the SPI-XG emulsion.

### 3.8. Antioxidant Activity

The antioxidant activities of the SPI-XG and SPI-CA W/O/W emulsions can be evaluated by measuring their capacity to scavenge ABTS and DPPH free radicals. The antioxidant activity of the emulsions for DPPH and ABTS increased from 45.76% and 46.39% (SPI) to 53.73% (XG 0.5%) and 55.47% (CA 0.5%) and 61.28% (XG 0.5%) and 64.78% (CA 0.5%), respectively. The SPI emulsion had the lowest antioxidant activity. The antioxidant activities of the emulsions were highest when the XG and the CA concentrations were 0.5%. The added XG and CA significantly (*p* < 0.05) reduced the number of free radicals (Figure 5B). Thus, the antioxidant activity of the SPI-XG/CA emulsion was enhanced. The SPI-XG and SPI-CA emulsions had a continuous phase with higher viscosity than the SPI emulsion. Thus, a denser film was formed at the phase interface and the contact area of their carboxyl and amino groups with free radicals increased. Additionally, the two-membrane and three-phase complex structure of the emulsion also reduced the loss of antioxidant substances and improved the antioxidant activity of the emulsion.

### 3.9. In Vitro Digestion

**Figure 6:**
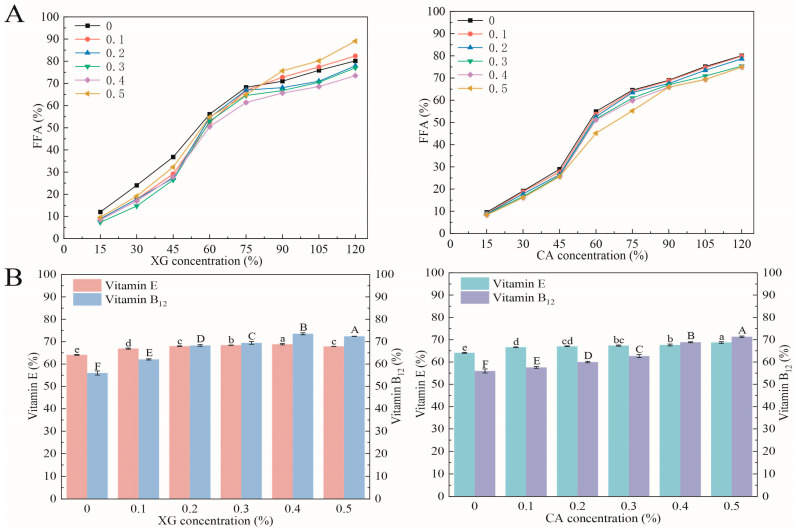
Release of free fatty acids (**A**) and bioavailability (**B**) of SPI-XG and SPI-CA emulsions with different polysaccharide concentrations. Different letters in the figures represent significant differences (*p* < 0.05).

All W/O/W emulsions exhibited a rapid increase in the FFA release rate during the first 60 min (Figure 6A). However, after this time, the rate began to slow down and then exhibited a gradual slow increase. During digestion, the SPI in the W_2_ of the droplets was rapidly replaced by bile salts and phospholipids, which promoted lipase attachment, caused the rapid attachment of lipase molecules at the surface of the oil phase, and facilitated droplet digestion [32]. The SPI emulsion had the highest FFA release rate, which indicated that it does not prevent droplet digestion sufficiently. In contrast, SPI-XG and SPI-CA complexes in the W_2_ increased the viscosity of the continuous phase of the SPI-XG and SPI-CA emulsions and increased the thickness of the SPI film at the phase interface. This inhibited lipid digestion and decreased the FFA release rate. The SPI-XG and SPI-CA W/O/W emulsions produced fewer FFAs, which was attributable to the ability of the complexes to resist displacement of bile salts, phospholipids, and lipases from the oil phase surface.

The bioaccessibilities of SPI-XG and SPI-CA W/O/W emulsions increased significantly (*p* < 0.05) as XG and CA concentrations increased (Figure 6B). The bioaccessibilities of the emulsions for vitamin B_12_ and vitamin E increased from 55.98% and 64.10% (SPI) to 73.53% (XG 0.4%) and 71.32% (CA 0.5%) and to 68.86% (XG 0.4%) and 68.74% (CA 0.5%), respectively. SPI emulsion had the lowest bioaccessibility. The bioaccessibilities of the emulsions were highest when the XG concentration was 0.4% and the CA concentration was 0.5%. This indicated that the added XG and CA effectively protected vitamin B_12_ and vitamin E. Bioaccessibility is the percentage of embedded vitamin B_12_ and vitamin E transferred to the mixed micelles during digestion, so that they can be absorbed in the intestine [33]. The bioaccessibility of the W/OW emulsion was dependent on changes in the FFA release rate. XG and CA retarded the breakdown of the emulsion and slowed down lipid digestion [34]. This resulted in higher bioavailability as more vitamin B_12_ and vitamin E could be retained in the micelles.

## 4. Conclusions

This study successfully prepared SPI-XG and SPI-CA co-delivery W/O/W emulsions. The particle sizes of the SPI-XG and SPI-CA emulsions decreased, and their zeta absolute potential values increased as XG and CA concentrations increased. The added XG and CA improved the SPI aggregation and increased the electrostatic repulsion, and thus, more SPI was adsorbed at the phase interface, and a denser film was formed at the interface. The emulsifying activity index and emulsifying stability index increased to 24.09 (XG 0.4%) and 14.00 (CA 0.5%) and to 151.08 (XG 0.4%) and 135.34 (CA 0.5%), respectively. The adsorbed protein content increased to 88.90% (XG 0.4%) and 88.23% (CA 0.5%), respectively. Moreover, the addition of XG and CA improved the rheological properties of the SPI-XG and SPI-CA emulsions, and more stable network structures were formed. The physicochemical stabilities, antioxidant activities, and encapsulation efficiencies of the W/O/W emulsion were improved. The encapsulation efficiencies of vitamin B_12_ and vitamin E were increased to 86.72% (XG 0.4%) and 86.47 (CA 0.5%) and 86.31% (XG 0.4%) and 85.78% (CA 0.5%), respectively. The antioxidant activity of the emulsions for DPPH and ABTS increased to 53.73% (XG 0.5%) and 55.47% (CA 0.5%) and 61.28% (XG 0.5%) and 64.78% (CA 0.5%), respectively. The SPI-XG and SPI-CA complexes increased the viscosity of the continuous phase to inhibit the surface displacement of digestive fluids and achieve slow release of vitamin B_12_ and vitamin E. The bioaccessibility of vitamin B_12_ and vitamin E increased to 73.53% (XG 0.4%) and 71.32% (CA 0.5%) and 68.86% (XG 0.4%) and 68.74% (CA 0.5%). The best properties of the emulsions were obtained at a 0.4% of concentration of XG and 0.5% of CA. Therefore, SPI-XG and SPI-CA W/O/W emulsions have a great potential for application as systems for the co-delivery of bioactive substances.

## Data Availability

The data presented in this study are available on request from the corresponding author.
